# Home Medical Care and Reduced Risk of Rehospitalization After Aspiration Pneumonia in an Elderly Japanese Population: A Nationwide Inpatient Database Study

**DOI:** 10.7759/cureus.99728

**Published:** 2025-12-20

**Authors:** Yasuyuki Fuseda, Sayuri Shimizu, Kiyohide Fushimi

**Affiliations:** 1 Department of Health Policy and Informatics, Institute of Science Tokyo, Tokyo, JPN; 2 Association of Medical Science, Department of Health Data Science, Yokohama City University, Yokohama, JPN

**Keywords:** administrative claims database, elderly people, home medical care, recurrent aspiration pneumonia, rehospitalization

## Abstract

Introduction

Aspiration pneumonia in elderly patients is a major public health burden associated with high mortality and frequent rehospitalization. In response to a rapidly aging society, Japan’s healthcare system is promoting a shift from hospital-centric treatment to home medical care. Although the benefits of home medical care have been established in settings such as palliative care, its effectiveness in preventing readmission of elderly patients recovering from aspiration pneumonia remains unclear. This study evaluated whether initiation of home medical care at discharge is associated with a lower risk of 365-day rehospitalization among elderly patients admitted for aspiration pneumonia.

Methods

This retrospective cohort study was conducted using data from Japan’s nationwide Diagnosis Procedure Combination database from April 1, 2015, to March 31, 2022. We identified patients aged ≥65 years who were hospitalized for their first episode of aspiration pneumonia (International Classification of Diseases, Tenth Revision (ICD-10) code: J69.0). We then excluded patients who were already receiving home medical care, experienced in-hospital death, or were discharged to other hospitals or nursing facilities. We conducted 1:4 propensity score matching with adjustment for age, sex, activities of daily living, and comorbidities to compare patients who received home medical care at discharge (home medical care group) and those who did not (control group). The primary outcome was all-cause rehospitalization to the same hospital within 365 days, analyzed using a Cox proportional hazards model. The secondary outcomes included rehospitalization for specific causes.

Results

Among 183,770 eligible patients, 12,454 in the home medical care group were matched to 49,816 in the control group. The baseline characteristics were well balanced after matching (standardized mean differences, <0.10 for all). The 365-day all-cause rehospitalization rate was lower in the home medical care group than in the control group (5,317 (42.7%) vs. 22,858 (45.9%); p < 0.001). Home medical care was associated with a lower risk of all-cause rehospitalization (hazard ratio (HR), 0.91; 95% confidence interval (CI), 0.89-0.94; p < 0.001). Regarding secondary outcomes, the home medical care group had lower risks of rehospitalization for aspiration pneumonia (HR, 0.92; 95% CI, 0.88-0.96; p < 0.001), urinary tract infections (HR, 0.84; 95% CI, 0.73-0.96; p = 0.009), and fractures (HR, 0.69; 95% CI, 0.56-0.85; p < 0.001).

Conclusions

The initiation of home medical care at discharge for elderly patients recovering from aspiration pneumonia was associated with a lower risk of 365-day all-cause rehospitalization. The risk reduction was particularly notable for readmission for aspiration pneumonia, urinary tract infections, and fractures. These findings support the potential value of home medical care, which has been expanded to manage an aging population and reduce the burden on acute-care hospitals.

## Introduction

Aspiration pneumonia, a bacterial lung infection caused by the aspiration of oropharyngeal and gastric contents, is a significant public health burden [1]. It accounts for 5% to 15% of community-acquired pneumonia cases and is associated with high mortality, with a reported 30-day mortality rate of 21% [2,3]. The clinical challenge is particularly pronounced among older adults aged ≥65 years. In Japan, a multicenter survey reported that 79% of hospitalized patients with pneumonia were aged >70 years, and aspiration pneumonia accounted for 42.8% of cases in this age group [4]. Patients at high risk for aspiration pneumonia are typically older and more prone to recurrent pneumonia and rehospitalization, creating a cycle of repeated hospital stays [2,5].

In response to this issue in a rapidly aging society, where individuals aged ≥65 years accounted for 29.0% of the total population in 2022 [6], Japan’s healthcare system is promoting a strategic shift from hospital-centric treatment to home medical care for elderly patients recovering from aspiration pneumonia [7-9]. However, although the demand for home medical care is rapidly increasing [10], evidence regarding its impact on specific clinical outcomes in this population remains limited.

The benefits of home medical care have been demonstrated in several clinical settings [11-13]. For example, home-based palliative care for patients with terminal illnesses has been shown to reduce readmissions and improve patient satisfaction [14,15]. Nevertheless, the effectiveness of this approach in preventing rehospitalization of elderly patients with a history of aspiration pneumonia remains unclear.

The present study evaluated whether the initiation of home medical care at discharge is associated with a lower risk of readmission among elderly patients hospitalized for aspiration pneumonia. The primary focus was on all-cause rehospitalization, with an exploratory analysis of cause-specific rehospitalization to investigate potential mechanisms. The findings are intended to serve as a foundation for future healthcare policy discussions.

## Materials and methods

Study design and data source

This retrospective cohort study used data from the Diagnosis Procedure Combination (DPC) database, a Japanese nationwide administrative claims and discharge abstract database that collects data for >7 million inpatients per year from approximately 1,200 hospitals [16,17]. The database contains detailed patient-level data, including demographic characteristics (age, sex, body mass index [BMI]), diagnoses encoded using International Classification of Diseases, Tenth Revision (ICD-10) codes, daily procedures, drug administrations, and clinical information such as activities of daily living (ADL) and levels of consciousness. A validation study showed good sensitivity and excellent specificity of diagnoses recorded in the database [18,19]. The database also contains information on whether home medical care was already in place at admission or was initiated at discharge. In addition, it allows identification of patients readmitted to the same hospital; however, readmissions to other hospitals cannot be tracked because of database limitations. For this study, we analyzed data for patients discharged between April 1, 2015, and March 31, 2022.

Participants

The inclusion criteria were age of ≥65 years and admission for a first episode of aspiration pneumonia (ICD-10 code: J69.0). The exclusion criteria were patients who were already receiving home medical care at admission, patients who experienced in-hospital death, patients who were transferred to other hospitals or discharged to nursing facilities, and patients with missing data at discharge. Patients discharged to nursing facilities were excluded because of differences in medical care provided in these facilities compared with that provided at home.

Variables and outcomes

The exposure was the initiation of home medical care at discharge. This information was extracted from Form 1 (clinical summary data) of the DPC database and included physician home visits, home nursing services, and home rehabilitation services. The primary outcome was all-cause rehospitalization to the same hospital within 365 days after discharge. The secondary outcomes included rehospitalization within 365 days for seven specific causes: aspiration pneumonia, urinary tract infections (ICD-10 codes: N10, N11, N12, N13.6, N15.1, N20.9, N39.0), heart failure (ICD-10 codes: I50.0, I50.1, I50.9, I11.0, I13.0, I13.2), fractures (ICD-10 codes: S02, S12, S22, S32, S42, S52, S62, S72, S82, S92, T02, T08, T10), chronic obstructive pulmonary disease (COPD) exacerbation (ICD-10 code: J44), dehydration (ICD-10 code: E86), and cerebral infarction (ICD-10 code: I63). Regarding comorbidities, a history of cerebral hemorrhage (ICD-10 code: I61), Parkinson’s disease (ICD-10 code: G20), and malignancy (ICD-10 codes: C00-C97) were identified using administrative claims data. Data on age, sex, Barthel Index, BMI, dementia severity, level of consciousness (Japan Coma Scale), smoking history, and ambulance use were extracted from Form 1 (clinical summary data).

Statistical analysis

We used propensity score matching to balance baseline characteristics between patients who received home medical care at discharge (home medical care group) and those who did not (control group). Missing data were handled using complete-case analysis. Propensity scores were estimated using a logistic regression model that included the following baseline variables: (1) hospital-level factor: average inpatient volume; (2) patient demographic characteristics and baseline status: age, sex, Barthel Index, BMI, dementia severity, level of consciousness (Japan Coma Scale), smoking history, and ambulance use at admission; (3) comorbidities: history of cerebral infarction, cerebral hemorrhage, Parkinson’s disease, COPD, heart failure, or malignancy; (4) in-hospital procedures: use of oxygen, tracheal intubation, antibiotics, vasopressors, or a central venous catheter; and (5) length of index hospitalization. Age was treated as a continuous variable, whereas all other covariates were treated as categorical variables; no interaction terms were included.

Patients were matched in a 1:4 ratio using nearest-neighbor matching with a caliper width of 0.2 standard deviations of the propensity score. Covariate balance after matching was assessed using standardized mean differences (SMDs), with an absolute SMD of >0.10 considered indicative of imbalance. In the matched population, the cumulative incidence of rehospitalization was evaluated using Kaplan-Meier survival curves. The time to rehospitalization was compared between the two groups using a Cox proportional hazards model, and the results were expressed as hazard ratios (HRs) with 95% confidence intervals (CIs). Absolute risk differences and their 95% CIs were calculated based on the cumulative incidence within 365 days using a linear regression model with robust standard errors clustered by matched pairs to account for the non-independence of the data. Statistical significance was defined as p = 0.05 (two-sided). All analyses were performed using Stata/SE version 17.0 (StataCorp, College Station, TX, USA), and propensity score matching was performed using the psmatch2 command.

## Results

Participant selection

In total, 696,124 patients with aspiration pneumonia were initially identified in the DPC database. Of these, we excluded 163,939 patients who were already receiving home medical care at admission, 601 patients with missing data on home medical care status, 107,968 patients who died during hospitalization, and 254,586 patients who were discharged to locations other than home. Consequently, 169,030 patients were included in the final analysis. This cohort was divided into patients who received home medical care at discharge (n = 14,446) and those who did not (n = 154,584). After 1:4 propensity score matching, the analytical groups comprised 12,454 patients in the home medical care group and 49,816 in the control group.

Baseline patient characteristics

The patients’ baseline characteristics before and after propensity score matching are summarized in Table 1. Before matching, significant differences were observed between the two groups. Patients in the home medical care group had lower ADL scores (proportion independent: 7.5% vs. 28.1%), lower BMI values (proportion with <17.9 kg/m²: 36.2% vs. 27.3%), and more severe dementia (proportion with severe dementia: 21.1% vs. 12.9%) than patients in the control group. After 1:4 propensity score matching, these differences were attenuated, and the baseline characteristics were well balanced between the two groups (all SMDs <0.10; variance ratio for age, 0.99).

**Table 1:** Baseline characteristics of patients before and after propensity score matching Data are presented as n (%) or median (interquartile range). SMD, Standardized Mean Difference; ADL, Activities of Daily Living; BMI, Body Mass Index; JCS, Japan Coma Scale; COPD, Chronic Obstructive Pulmonary Disease.

	Before matching			After matching		
Variable	Control	Home medical care		Control	Home medical care	
	n = 154,584	n = 14,446	SMD	N = 49,816	N = 12,454	SMD
	(%)	(%)				
Sex (male)	63.1	61.9	0.037	60.6	61.5	0.019
Age, years	84 (78-89)	85 (79-90)	0.093	85 (79-90)	85 (79-90)	0.015
ADL at discharge			0.72			0.011
0	19.8	44		43.9	44	
5-50	29.8	34.1		34.4	34.1	
55-95	22.2	14.4		14.5	14.3	
100	28.1	7.5		7.2	7.6	
Body mass index, kg/m^2^			0.175			0.005
<17.9	27.3	36.2		37.1	36.7	
18.0-19.9	18.3	18.1		18.2	18.3	
20.0-21.9	16.9	14.1		14.1	14	
22.0-24.9	17	12.5		12.3	12.6	
≧25.0	20.6	19		18.4	18.4	
Dementia			0.35			0.017
No dementia	47.3	32.1		31.3	31.8	
I or II	39.8	46.7		47.3	46.5	
III, IV, M	12.9	21.1		21.4	21.7	
smoking			0.04			0.014
Non-smoker	59.8	61.1		62.1	61.7	
Ex- or current smoker	40.2	38.9		37.9	38.3	
JCS score at discharge			0.312			0.007
Alert	81.4	69.5		69.6	68.9	
Dizziness	16.9	25.3		24.9	25.6	
Somnolence	1.5	4.6		4.8	4.8	
Coma	0.2	0.6		0.6	0.7	
Use of ambulances (Yes)	48.1	51.4	0.082	52.6	52.1	0.004
Length of stay, days			0.651			0.007
≦7	11.7	4		3.5	3.9	
8-28	68.6	48.2		48.6	48.3	
29-84	18	40.6		41.2	40.7	
85-365	1.7	7.1		6.6	7.1	
>365	0	0.1		0.1	0.1	
Cerebral infarction	4.1	4.7	0.029	4.5	4.6	0.014
Cerebral hemorrhage	0.6	0.7	0.002	0.7	0.6	0.014
Parkinson disease	4.7	7.8	0.129	7.6	7.8	0.011
COPD	4.7	4.9	0.006	4.7	4.8	0.022
Heart failure	15.4	15.4	0.005	15.4	15.4	0.003
Malignancy	16.1	18.1	0.047	17.7	18.1	0.007
Oxygen therapy	60.7	69.3	0.187	69.4	69.2	0.017
Intubation	1.1	1.6	0.047	1.5	1.7	0.016
Central venous cannulation	2.6	6.8	0.203	6.7	6.9	0.002
Antibacterial drug	97	96.6	0.013	98.1	98	0.006
Vasopressor	3.4	4.9	0.084	4.9	5.1	0.008
Tertiles of daily inpatient volume			0.04			0.016
1st tertile (Low)	33.4	35.7		35.7	35.9	
2nd tertile (Medium)	33.3	31.8		31.1	31.9	
3rd tertile (High)	33.4	32.5		33.2	32.1	

Primary and secondary outcomes

**Figure 1:**
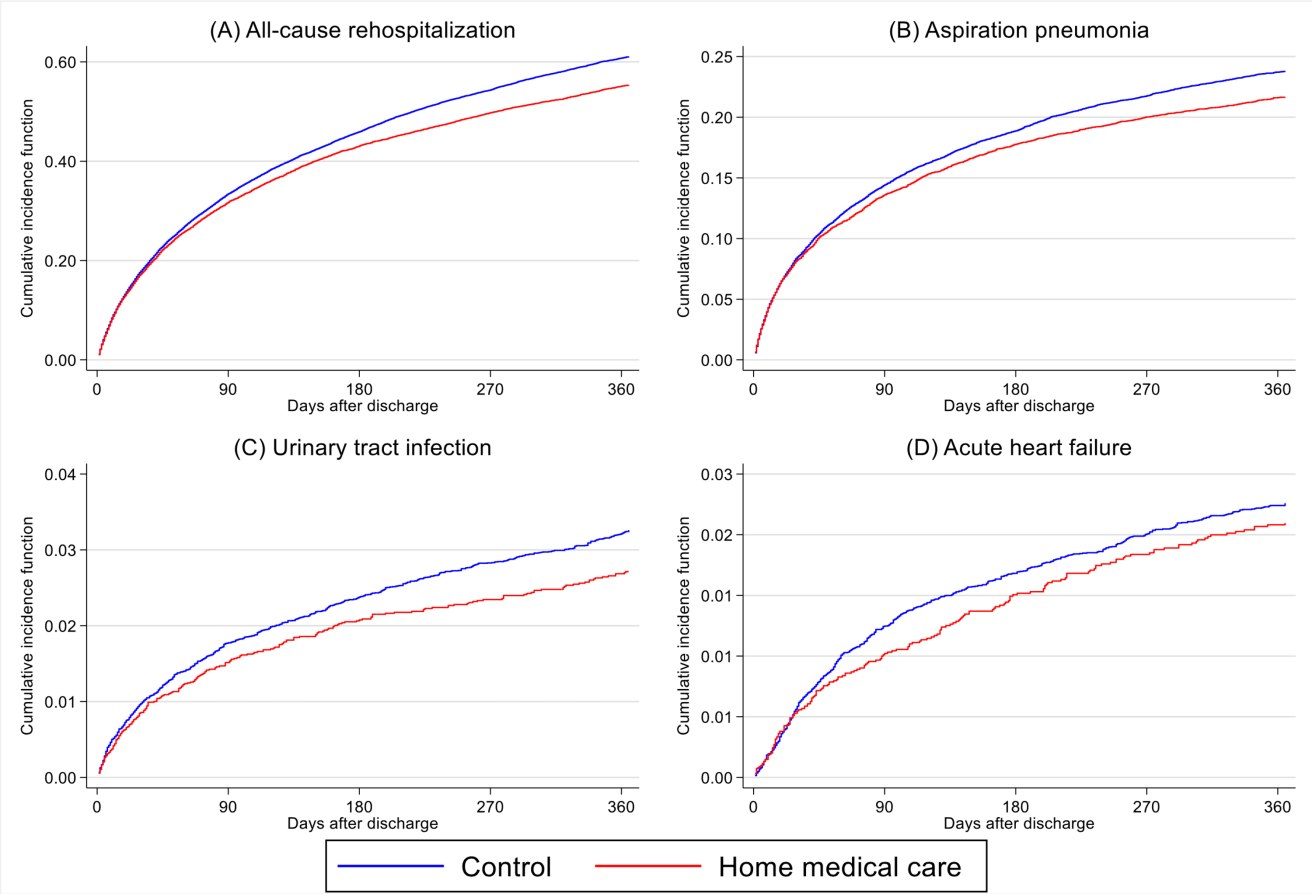
Cumulative incidence curves for 365-day rehospitalization in the propensity score-matched cohort (A-D) Cumulative incidence curves for 365-day rehospitalization comparing the home medical care group (red line) and the control group (blue line). The panels show: (A) All-cause rehospitalization, (B) Aspiration pneumonia, (C) Urinary tract infection, (D) Acute heart failure

**Figure 2:**
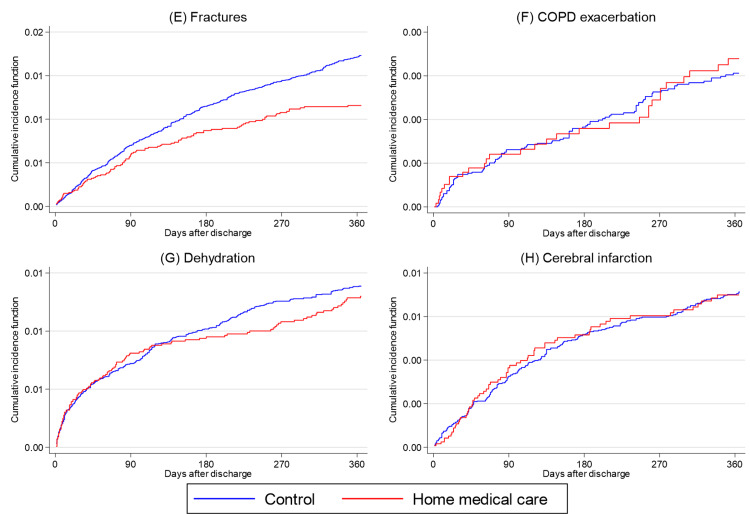
Cumulative incidence curves for 365-day rehospitalization in the propensity score-matched cohort (E-H) Cumulative incidence curves for 365-day rehospitalization comparing the home medical care group (red line) and the control group (blue line). (E) Fractures, (F) COPD (chronic obstructive pulmonary disease) exacerbation, (G) Dehydration, and (H) Cerebral infarction.

For the primary outcome, the 365-day all-cause rehospitalization rate was lower in the home medical care group than in the control group (5,317 (42.7%) vs. 22,858 (45.9%); absolute difference, 3.2%; p < 0.001) (Table 2). Figures 1, 2 show the cumulative incidence curves for rehospitalization. In the Cox proportional hazards model, receipt of home medical care was associated with a lower risk of rehospitalization (HR, 0.91; 95% CI, 0.89-0.94; p < 0.001).

**Table 2:** Risk difference for 365-day rehospitalization by cause Data are presented as n (%). RD, Risk Difference; CI, Confidence Interval; COPD, Chronic Obstructive Pulmonary Disease.

	Control		Home medical care		RD		
	N = 49,816		N = 12,454				
	n	(%)	n	(%)	(%)	95% CI	
All-cause rehospitalization	22,858	45.88	5,317	42.7	3.19	2.14	4.25
Aspiration pneumonia	9,168	18.4	2,138	17.17	1.24	0.42	2.05
Urinary tract infection	1,215	2.44	262	2.1	0.34	0.02	0.65
Acute heart failure	834	1.67	195	1.57	0.11	-0.16	0.37
Fractures	607	1.22	108	0.87	0.35	0.15	0.56
COPD exacerbation	110	0.22	30	0.24	-0.02	-0.12	0.08
Dehydration	522	1.05	125	1.00	0.04	-0.17	0.26
Cerebral infarction	259	0.52	64	0.51	0.01	-0.14	0.15

Regarding secondary outcomes (Table 3), the home medical care group had a lower risk of rehospitalization for aspiration pneumonia (HR, 0.92; 95% CI, 0.88-0.96; p < 0.001), although the absolute risk reduction was modest. Lower risks of rehospitalization were also observed for urinary tract infections (HR, 0.84; 95% CI, 0.73-0.96; p = 0.009) and fractures (HR, 0.69; 95% CI, 0.56-0.85; p < 0.001). By contrast, no significant differences were observed between the two groups in the risk of rehospitalization for heart failure (HR, 0.92; 95% CI, 0.79-1.07; p = 0.279), COPD exacerbation (HR, 1.10; 95% CI, 0.73-1.64; p = 0.660), dehydration (HR, 0.95; 95% CI, 0.78-1.15; p = 0.576), or cerebral infarction (HR, 0.98; 95% CI, 0.75-1.29; p = 0.910).

**Table 3:** Hazard ratios for 365-day rehospitalization by cause in the propensity score-matched cohort Results from the Cox proportional hazards model. The control group served as the reference category. HR, Hazard Ratio; CI, Confidence Interval; COPD, Chronic Obstructive Pulmonary Disease.

		HR	95% CI	P value
All-cause rehospitalization	control	Reference		
	Home medical care	0.91	0.89-0.94	<0.001
Aspiration pneumonia	Control	Reference		
	Home medical care	0.92	0.88-0.96	<0.001
Urinary tract infection	Control	Reference		
	Home medical care	0.84	0.73-0.96	0.009
Acute heart failure	Control	Reference		
	Home medical care	0.92	0.79-1.07	0.279
Pathological fractures	Control	Reference		
	Home medical care	0.69	0.56-0.85	<0.001
COPD exacerbation	Control	Reference		
	Home medical care	1.1	0.73-1.64	0.66
Dehydration	Control	Reference		
	Home medical care	0.95	0.78-1.15	0.576
Cerebral infarction	Control	Reference		
	Home medical care	0.98	0.75-1.29	0.91

## Discussion

In this nationwide retrospective cohort study of elderly patients with aspiration pneumonia, initiation of home medical care at discharge was associated with a lower risk of all-cause rehospitalization within 365 days. In particular, reduced risks of rehospitalization for specific causes, including aspiration pneumonia, urinary tract infections, and fractures, were observed, although the absolute differences were small.

In this study, initiation of home medical care at discharge was shown to lower the risk of rehospitalization among elderly patients in Japan. This finding is consistent with results from international studies by Brumley and colleagues, who used different methods, including a comparative study and a randomized controlled trial, to show that home medical care was associated with reduced hospital use [14,15]. We acknowledge, however, that those studies focused on palliative care populations, which differ from the elderly population recovering from aspiration pneumonia in the present study. A plausible explanation for our findings is that home medical care may provide continuous management, allowing emerging problems to be addressed before they require hospital-based care, thereby reducing pressure on the acute-care hospital system.

The findings regarding cause-specific rehospitalization offer insight into potential mechanisms by which home medical care may benefit patients. The reduced risk of readmission for aspiration pneumonia and urinary tract infections suggests that the ability to manage common infections in the home setting (e.g., through early initiation of antibiotic treatment) may play an important role. Home medical care teams frequently respond to early signs of clinical deterioration, such as fever, general malaise, and reduced oral intake, which often precede hospital admission. Moreover, the lower risk of rehospitalization for fractures may reflect the impact of environmental assessments and fall prevention strategies implemented during home visits. Taken together, these observations suggest that home medical care may reduce rehospitalization related to common infections and environmental risks, although this interpretation remains speculative. This finding is also clinically relevant because it aligns with the general preference for home-based care among the Japanese population [20].

Several limitations of this study should be considered. First, rehospitalization could only be identified if it occurred at the same hospital as the index admission because readmissions to other hospitals were not captured in the database. As a result, the incidence of rehospitalization is likely underestimated. Furthermore, if the likelihood of readmission to a different hospital differed between patients who did and did not receive home medical care, this limitation could have biased the estimated association. Second, death after discharge represents a competing risk because patients who died at home could not undergo readmission; this may have influenced the estimated rehospitalization rates. Third, as with other studies using administrative databases, some clinically relevant data were unavailable. In particular, the A-DROP score was missing for 83% of eligible patients, and detailed assessments of swallowing function were not available. These limitations prevented a full adjustment for pneumonia severity and may have resulted in residual confounding. Furthermore, because of the structure of the DPC database, we could only identify whether home medical care was initiated, not the frequency or specific content of services provided, limiting the replicability of the intervention. Diagnostic misclassification is also possible. Fourth, we excluded a substantial number of patients, particularly those discharged to nursing facilities, to focus on patients returning home. These exclusions may have introduced selection bias and limited the generalizability of our findings to the broader population of patients with aspiration pneumonia. Fifth, we did not adjust for multiple comparisons for secondary outcomes; therefore, the risk of type I error cannot be ruled out, and the results for these outcomes should be interpreted as exploratory. Finally, although the reduction in rehospitalizations was statistically significant, the effect size was modest (HR, 0.91). This finding should be interpreted with caution because unmeasured residual confounding may have influenced the results despite extensive adjustments. In light of these limitations, prospective studies are warranted to confirm the present findings and achieve better control of confounding factors.

## Conclusions

Our analysis of data from a large nationwide database suggests that initiation of home medical care is associated with a lower risk of 365-day rehospitalization among elderly patients recovering from aspiration pneumonia, although the absolute risk reduction is modest. The reduced risk was primarily observed for readmission for aspiration pneumonia, urinary tract infections, and fractures. As one of the few studies to quantify this association in Japan, our findings provide important evidence to inform future discussions on the role of home medical care in healthcare strategies for an aging population.
